# Activity Detection of Paralympic Athletes with Lower Limb Running-Specific Prosthesis During Extended Periods of Time: Software Development and Preliminary Validation

**DOI:** 10.3390/s26010097

**Published:** 2025-12-23

**Authors:** Mirco Tioli, Isotta Bernardoni, Maria Grazia Santi, Roberto Di Marco, Giuseppe Marcolin, Nicola Petrone, Andrea Giovanni Cutti

**Affiliations:** 1 Department of Industrial Engineering, University of Padova, 35131 Padua, Italy; mircotioli98@gmail.com (M.T.); m.santi@inail.it (M.G.S.); nicola.petrone@unipd.it (N.P.); 2 D-ITET, ETH Zurich, Gloriastrasse 35, 8092 Zürich, Switzerland; ibernardoni@student.ethz.ch; 3 Centro Protesi INAIL, Via Rabuina 14, 40054 Vigorso, Italy; r.dimarco@inail.it; 4 Department of Biomedical Sciences, University of Padova, 35131 Padua, Italy; giuseppe.marcolin@unipd.it

**Keywords:** IMU, lower-limb amputation, Paralympics, running prosthesis, activity monitoring, stride counting, sprinting, triathlon

## Abstract

Monitoring the activities of athletes with lower-limb amputations who use running-specific prostheses is essential for evaluating their training regimes, as well as the effectiveness and mechanical fatigue wear of their prostheses over time. Recent advancements in Inertial Measurement Units (IMUs) and activity detection algorithms offer new opportunities for objective assessment, but their application in Paralympic sports remains unexplored. The aims of this work were to design and implement an innovative protocol and analytical software for *short-term* and *long-term* activity detection of athletes with transtibial and transfemoral amputation and then test its validity on a sample of elite Paralympic runners and triathletes. Overall, the ability of the model to detect activities presented an accuracy of 98%, and the error in the stride counting for all activities fell within a 1% margin.

## 1. Introduction

Sport therapy is a significant component in the rehabilitation of individuals who have suffered a limb amputation, thanks to its beneficial contributions to both their physical and psychological well-being [1,2,3]. This approach has gained significant traction since the pivotal works of the 1950s [4,5], inspiring some individuals to become professional athletes and many to adopt an active and participatory lifestyle. The Paralympic Games are now recognized as a major sporting event [6].

In this context, monitoring the activities of persons with amputations using sport-specific prostheses can support the assessment of health condition, evaluate training regimes, and optimize prostheses safety and effectiveness [7,8,9,10]. On this regard, the availability of long-term assessments can support the definition of standardized cyclic mechanical bench tests on sport-specific prosthesis sockets and running feet, which are currently lacking.

In recent years, the development of smaller, more precise sensors, such as Inertial Measurements Units (IMUs), has allowed for continuous monitoring over extended periods of time, leading to improvement in the sport analysis field [11,12]. Additionally, advances in machine learning led to new algorithms for pattern recognition [13]. Despite these advancements, the literature addressing the specific application of these tools on athletes with amputations remains extremely limited.

The application of IMU-based activity monitoring for able-bodied individuals is well-known [14,15]. However, existing algorithms may struggle in accommodating signal morphology induced by gait adaptations of prosthesis users, and signal variations specific to sports activities. While step-counting in walking has been widely studied in persons with amputations [16,17,18,19,20], it is only recently that studies on activity detection have become available [21,22,23,24,25,26]. However, most of these studies relied on the combination of both accelerometers and gyroscopes [24,25,26], limiting battery life [24,25,26], and involved only few participants with amputations in a sample of able-bodied individuals [21,24,25] or persons with powered/robotic prostheses [22,23]. Moreover, the goal of these studies was to detect whether the participant was standing, sitting, lying down or walking horizontally or up/down the stairs [21,22,23,24,25], while jogging and sprinting activities have never been part of the recognized pool of activities [21,22,23,24,25,26].

This study aimed to address these limitations by designing and validating an activity detection and stride counting algorithm using data from a single, low-sampling frequency triaxial accelerometer, for long-term activity monitoring of track-and-field and triathlon athletes with lower-limb amputations. Specifically, we hypothesized that a triaxial accelerometer sampling at 12.5 Hz could be matched with an algorithm reaching a high subject-specific accuracy, with the possibility to record up to 140 days. The solution was then applied to a pool of seven elite Paralympic athletes during the 40 days before the 2024 Paris Paralympic Games.

## 2. Materials and Methods

### 2.1. Participants

Eight elite athletes of the Italian Paralympic team (three females, five males; age: 29 ± 11 years; four with TT unilateral, three with TF unilateral, one with TT bilateral amputations–Table 1) were recruited and gave informed consent to participate prior to data collection. The study protocol was approved by the bioethical committee of the Department of Biomedical Sciences of the University of Padua (HEC-DSB/6-2024).

Exclusion criteria were the presence of painful neuromas on the distal end of the residual limb and cognitive impairments. Dropout criteria included the voluntary withdrawal of the subject, the loss of suitability to continue the experimental activities as evaluated by the principal investigator, device malfunctions, loss of medical clearance for sport practice and abandonment of sport practice.

### 2.2. Equipment

Accelerometric data were recorded with an Axivity AX6 (Axivity Ltd., Newcastle upon Tyne, UK), featuring a triaxial Micro-Electro-Mechanical System (MEMs) accelerometer: the device size is 23 × 32.5 × 8.9 mm, it weighs 11 g, is IP68 certified, and is equipped with an on-board non-volatile 1024 MB flash memory and a real-time quartz clock. The AX6 operates at selectable frequencies ranging from 12.5 Hz to 1600 Hz, with each increment doubling the previous frequency. For this study, AX6 was set to operate at 12.5 Hz and only triaxial accelerations were collected (accelerometer range: ±16 g). Based on the AX6 manual, this sensor configuration is suitable for about 140 days of continuous use [27].

In addition to the IMU, video recordings were used to serve as a reference for activity tracking and stride counting. Specifically, all videos were captured using a Panasonic JVC Everio (Kadoma, Osaka, Japan) hand-held camera, saving files in an uncompressed MKV format sampled at 25 Hz.

### 2.3. Software Implementation

All algorithms were implemented using Python 3.9.21 (Python Software Foundation, Wilmington, DE, USA), with Anaconda 2024.10 (Anaconda Inc., Austin, TX, USA) to manage dependencies and environments, and Spyder (community-developed) as an Integrated Development Environment. A specific environment was created, composed of the following libraries: NumPy, matplotlib, pandas, csv, cv2, tkinter, PIL, SciPy, sklearn, concurrent, seaborn, openpyxl, collections, decimal, PyWavelets, TensorFlow, datetime and math. Apps were developed, ensuring that there was no need for the user to interact with the script, only to interact with Graphical User Interfaces (GUIs), which were implemented using the functionalities of the tkinter library.

### 2.4. Definition of Activities and Motion Intervals

For this study, we defined four different activities to be categorized, identified and monitored, aimed at covering the main activities that an athlete may perform during a training session:

1. *Stop*: the athlete is either sitting or standing still, in general performing an activity that does not cause any relevant stress to the prosthetic device.
2. *Walk*: the athlete is walking, with the presence of the double support phase during the gait cycle.
3. *Jog*: for track-and-field athletes, this activity indicates the longer-distance lower-intensity running aimed at warming-up before a race.

For triathlon athletes, this activity includes all the running activities lacking the double support phase and is not limited to the warming-up.

4. *Sprint*: for track-and-field athletes, this activity indicates a trial where the athlete is starting from a still position and then runs with the aim to cover a predefined short straight distance, typically between 60 m and 100 m, as quickly as possible. The acceleration and deceleration phase are typically longer than the steady state velocity phase.

For triathlon athletes, this activity was not recorded, as it is not a usual part of their training regimes.

Moreover, we defined Motion Interval (MI) as a burst of the same activity with the following features:

- *Stop*: a time interval with a duration of at least 20 s;
- *Walk* and *Jog*: a time interval containing between 10 and 15 strides;
- *Sprint*: the time interval between the athlete’s start from a still position to maximum speed and initial deceleration.

### 2.5. Acquisition Protocol

A *short-term* session consisting of a typical training session was recorded for each athlete for the purpose of algorithm calibration and validation. This was followed by a *long-term* session of around 40 days prior to the 2024 Paralympic Games, to exploit the solution to monitor the activities of the athlete during a highly intense training period.

**Figure 1:**
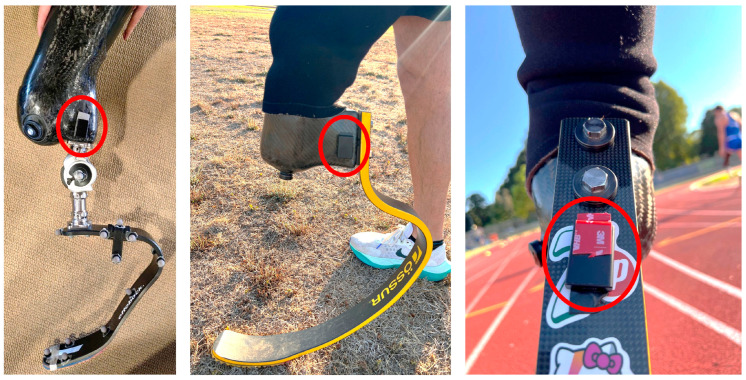
Different positioning of AX6 for different devices. On the left, a TF setup. In the middle, a TT standard setup. On the right, the custom positioning on the TT foot for athlete 005.

Specifically, during all sessions, one AX6 sensor was placed on the prosthesis. As a general guideline, it was positioned laterally on the socket for both TT and TF amputations, with one of the axes vertical to the ground when the subject was standing upright. However, some deviations were needed, depending on the athlete, since the socket shape is custom-made based on the conformation of the residual limb. Moreover, in selecting the sensor position, we excluded any obstruction of movement to the athletes or to the prosthetic knee, ensuring sensor stability relative to the socket by using double-sided tape. These criteria led to the development of a second possible configuration for TT amputations where the sensor is positioned on the proximal part (non-deformable) of the running prosthesis foot, resulting in about 10° of sensor inclination relative to the gravity vector while standing (Figure 1). For this study, this extra configuration was needed only for athlete 005.

During the *short-term* session, athletes were tracked with AX6 and the hand-held video camera during their typical individual training session (about 1–1.5 h) to record all the six activities defined in Section 2.4. Before the recordings, the camera operator identified a spot in the training facility to collect all videos, maintaining a distance of at least 10 m from the athlete. During recordings, the operator visually followed the athlete during training, using the camera zoom to allow for a clear identification of the activity and strides taken by the subject. The training sessions were altered as little as possible compared to standard practice, but ensuring the collection of sufficient signals for the identification of at least the following MIs for each athlete:

- Eight of *Stop*;
- Eight of *Walk*;
- Eight of *Jog*;
- Five of *Sprint*.

For *long-term* monitoring, each participant wore the AX6 positioned during the *short-term* session and kept it on the prosthesis during daily training sessions. The AX6 was removed before the competition to avoid possible infringements of the International Paralympic Committee regulations.

### 2.6. Algorithm Workflow

**Figure 2:**
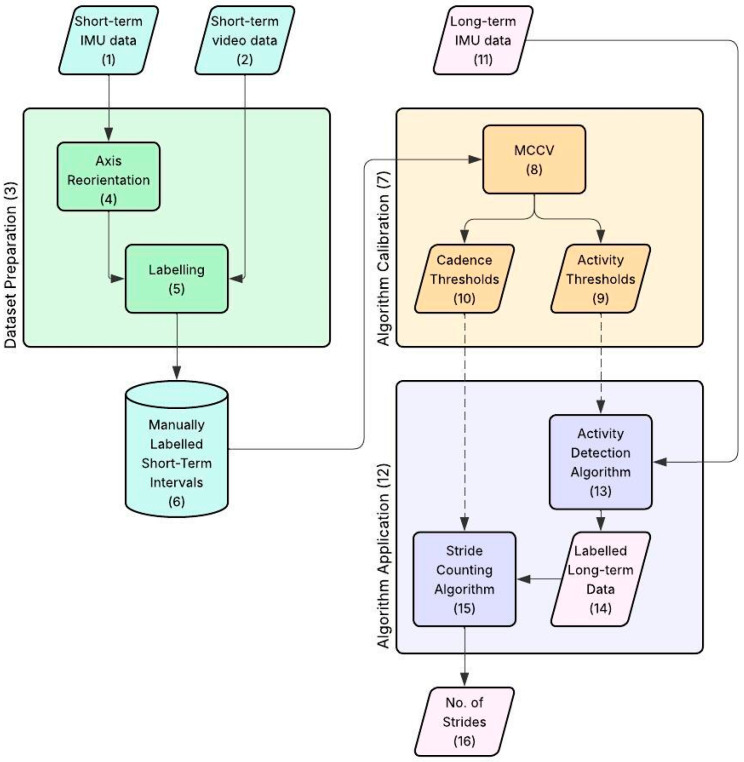
Overview of the whole workflow: in light blue (blocks 1–2, 6), the data and data structures obtained from the *short-term* acquisition. In pink (blocks 11, 14, 16), the data and results obtained from the *long-term* monitoring. In green (blocks 4–5), the dataset preparation. In orange (blocks 8–10), the algorithm calibration. In purple (blocks 13, 15), the algorithm application. Each block of the workflow is identified by a number from 1 to 16.

The task of activity monitoring and stride counting was addressed by first designing and implementing an algorithm for activity detection that segments the signal into MIs, followed by another algorithm for stride counting, to be applied within the same type of activity. The overall algorithm workflow is reported in Figure 2.

Hereinafter, a block numbered “B” within a figure “F”, will be referred to as “Block F.B”. IMU data from the *short-term* (Block 2.1) and the *long-term* (Block 2.11) acquisitions, together with the *short-term* video data (Block 2.2), are taken as an input to generate as output *long-term* labelled data with the corresponding activities (Block 2.14) and the total number of strides counted for each activity (Block 2.16).

The process can be divided into three main stages that are briefly described here and in more detail in the corresponding sections:

1. *Dataset Preparation* (Section 2.7): this stage (Block 2.3) creates the labelled database used for calibration and performance evaluation. Specifically, the *short-term* MIs are manually labelled (Block 2.5) by the user based on video data, to obtain the ‘gold standard’ database (Block 2.6). This stage also contains a pre-processing step in which the axes of the IMU signals are re-oriented (Block 2.4) for consistency among athletes.
2. *Algorithm Calibration* (Section 2.8): This stage (Block 2.7) calculates the parameters needed to calibrate the models for the specific subject. The main bulk of this stage is composed by the *Monte Carlo Cross Validation (MCCV)*, which is a cycle with 50 iterations (Block 2.8) used to estimate the subject-specific thresholds (Block 2.9) and cadences (Block 2.10), together with the subject-specific estimation of the algorithm classification and counting errors.
3. *Algorithm Application* (Section 2.9): This is the final stage (Block 2.12) of the procedure, where the algorithm is applied to the *long-term* data (Block 2.11). It is composed of two main blocks:
  - *Activity Detection Algorithm* (Block 2.13): using the thresholds from Block 2.9, it performs an automatic identification of the activities on the *long-term* data, giving as an output the labelled *long-term* data (Block 2.14);
  - *Stride Counting Algorithm* (Block 2.15): using the cadences from Block 2.10, it performs automatic counting of the total strides for each activity on the labelled *long-term* data (Block 2.14), giving as an output the number of strides performed over the whole acquisition for each kind of activity (Block 2.16).

### 2.7. Dataset Preparation

**Figure 3:**
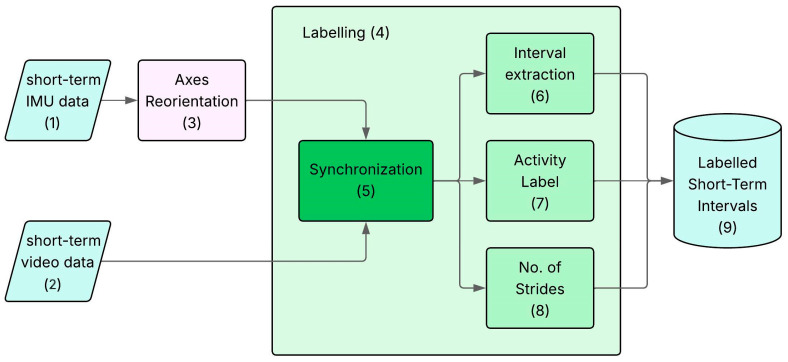
Representation of the workflow of the dataset preparation. In light blue, the in input and output data (blocks 1, 2, 9). In purple (block 3), the pre-processing. In dark green (block 5), the synchronization of the two signals. In light green (blocks 6–8), the labelling procedures manually performed by an operator.

The dataset preparation process is described in detail in Figure 3.

**Figure 4:**
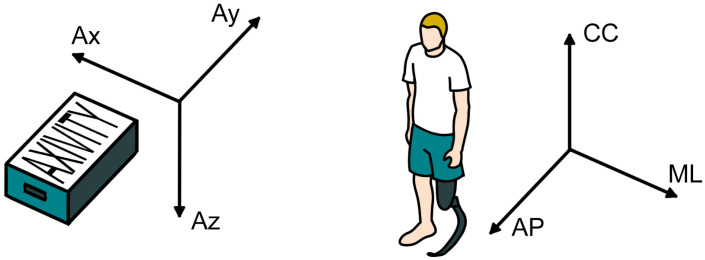
Axes reference system of the AX6 and of the human body after reorientation to obtain consistency across subjects. AP: anterior–posterior; CC: caudal–cranial; ML: medio-lateral.

The first step consists of reorienting the sensor axes (Block 3.3), as shown in Figure 4. Due to variations in sensor placement among different participants, straight comparison of accelerations across columns is highly misleading. To ensure comparability across subjects, raw accelerations were realigned to a standardized anatomical reference frame across subjects, while preserving the first column for the time step: the anterior–posterior (AP) axis was assigned to the second column, the caudal–cranial (CC) axis to the third, and the medio-lateral (ML) axis to the fourth column.

After the axis reorientation, the main block of the dataset preparation procedure is the labelling (Block 3.4). Considering the *short-term* session, the aim of the block was the creation of a database of MIs (Block 3.9), each tagged with the label indicating the activity and the number of strides, manually identified and counted based on the video recording used as gold standard. Specifically, every frame of the IMU data was paired with a frame of the video recordings aligning the time series (Block 3.5). To do so, the starting time of the IMU signal and of the video recordings were synchronized, based on the global timing collected by both measurement systems. The synchronization enabled the simultaneous visualization of IMU signals and video footage within a custom App, allowing the user to segment the signal into MIs (Block 3.6). Within each MI, the operator classified the activities (Block 3.7) and manually counted the number of strides (Block 3.8).

### 2.8. Algorithm Calibration

**Figure 5:**
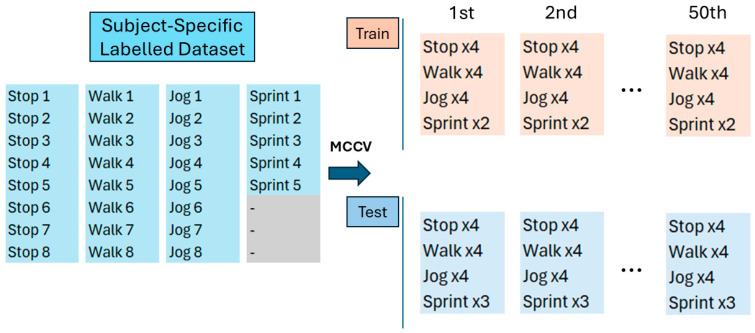
Structure of the stratified MCCV database: from the full labelled database (**left**) to 50 Train (**up right**) and 50 Test (**down right**) files.

To obtain subject-specific thresholds and cadences to calibrate the activity detection algorithm and the stride counting algorithm, a stratified MCCV [28] was performed, taking as inputs the labelled dataset. The output of the MCCV is a new dataset, which is graphically summarized in Figure 5.

The MCCV block performed the random partitioning of the *short-term* labelled dataset into 50 training and testing sets. This approach allowed for the creation of multiple Training and Testing Datasets starting from a single database, expanding the dataset to make a subject-specific threshold estimation and providing information regarding the accuracy (error) of the algorithms in the detection of different sport activities at subject-specific levels.

**Figure 6:**
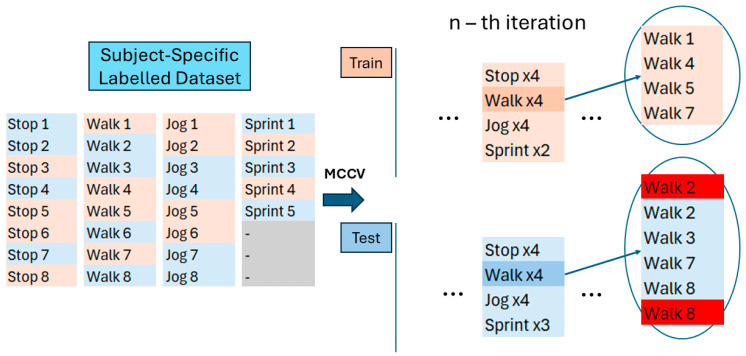
Zoom in of the structures generated by a generic n-th iteration of the MCCV. In light orange, the MIs randomly chosen to be part of the Train Dataset, while in blue, the MIs part of the Test Dataset. In bright red, the position of the doubled MIs at the edges of each category of the Test Dataset.

Figure 6 reports a zoom-in on Figure 5, to describe the structure of the datasets generated at the n-th iteration of MCCV.

Specifically, at the n-th iteration of the MCCV, four MIs for *Stop*, *Walk* and *Jog* and two MIs for *Sprint* were randomly extracted and assigned to the corresponding iteration of the Train Dataset, while the remaining MIs (four for *Stop*, *Walk* and *Jog* and three for *Sprint*) were assigned to the Test Dataset. Each dataset was composed of the concatenation of all the MIs assigned to it, keeping all the MIs with the same activity adjacent. The order of the activities is the following and was kept constant for both train and test sets: *Stop*, *Walk*, *Jog* and *Sprint*. The MIs at the edges of each category of the Test Dataset were doubled and marked to be ignored during the testing analysis, as the transition from the activities would be sudden and unnatural and would generate an artificial error.

#### 2.8.1. Activity Thresholds Estimation

**Figure 7:**
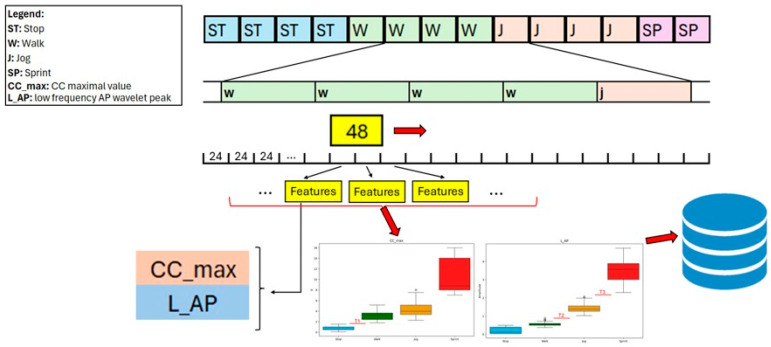
Zoom in of the procedure to extract the thresholds performed during the n-th iteration of the MCCV. While the window slides over the signal, each subinterval of 24 frames was assigned a set of two features. Then, by calculating the boxplots to show the distribution of the two features across the whole signal, three thresholds were identified and stored in a database. The box plots reported in the figure can be visualized in detail in Figure 9.

To calibrate the activity detection algorithm for the specific subject, three thresholds must be identified. These thresholds were calculated for each n-th iteration of the MCCV and then stored. The procedure used to obtain the thresholds is based on the use of a 48-frame sliding window with an overlap of 24 frames, that scrolls through the Train Database finding a set of features for every 24 frames of signal. The whole procedure can be seen in Figure 7.

The two features used to discriminate the activities, namely CC_max and L_AP, are presented in Table 2 and were calculated based on analyses in the time and frequency domain. The selection of these features was inspired by the fact that higher intensity activities might differ both in terms of acceleration (amplitude) of the signal and in terms of its frequency.

The parameter CC_max is obtained from the time-domain analysis of the signal. It corresponds to the maximum amplitude on the CC axis and is calculated as follows:

(1) $${MaxAmp}_{k}={max}_{1\leq i\leq N}\left|x_{k+i}\right|,$$

where *x* is the acceleration value at the frame i, with i varying from 1 to 48, indicating the possible frames in a k-window. Using this process, a value for the parameter was obtained for every window.

The parameter L_AP is instead a result of the frequency domain analysis. For this analysis, every acceleration axis of every 48-frame window was subject to a Continuous Wavelet Transform (CWT). In particular, the Morlet Complex Wavelet (cmor1.0–0.5 from Python library PyWavelets) was used based on its effectiveness with oscillatory quasi-stationary signals, where 1.0 represents the bandwidth of the wavelet and 0.5 specifies its centre frequency. The formula used for the CWT is the following:

(2) $$W\left(a,b\right)=\frac{1}{\sqrt{a}}\int_{-\infty }^{+\infty }x\left(t\right)\psi^{*}\left(\frac{t-b}{a}\right)dt,$$

where

1. *x*(t): input signal in time domain (accelerometer signal in AP axis, with a length of 48 frames);
2. *ψ*(t): mother wavelet function;
3. ψ*: complex conjugate of the mother wavelet;
4. *a*: scale parameter (inversely proportional to frequency);
5. *b*: translation (time shift) parameter.

The mother wavelet parameters were chosen to ensure a suitable balance between time and frequency localization for analyzing low-frequency components of accelerometer signals. The mother wavelet function was calculated as follows:

(3) $$\psi \left(t\right)=\pi^{-\frac{1}{4}}e^{j\omega_{0}t}e^{\frac{{-t}^{2}}{2}},$$

where

1. $\omega_{0}$: central frequency (related to the parameters ‘1.0–0.5’);
2. $e^{\frac{{-t}^{2}}{2}}$: provides time localization;
3. $e^{j\omega_{0}t}$: sinusoidal oscillation for frequency localization.

**Figure 8:**
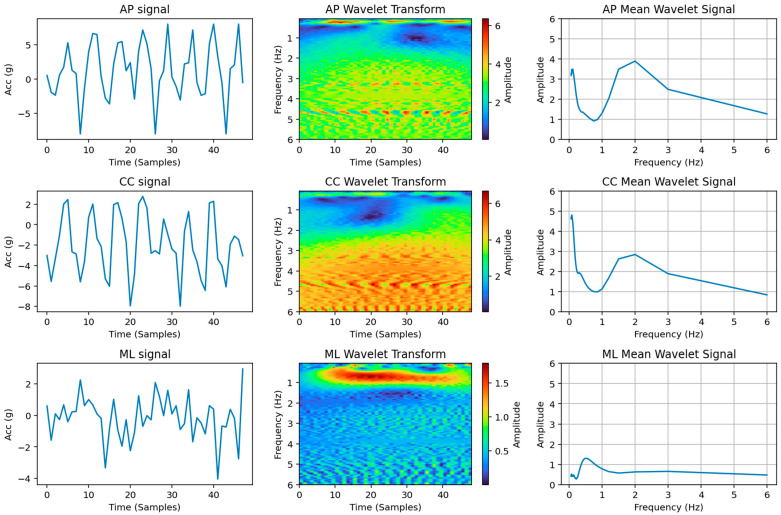
Example of the wavelet analysis of a *Sprint* MI from subject 006: on the left, the acceleration signal on the three axes; in the middle, the visualization of the wavelet transforms; and on the right, the mean frequency pattern.

After the wavelet transform of a window was calculated, the mean value of the 48 frames of the window was obtained for every frequency band. This gave, as a result, the mean behaviour of the wavelet transform inside the desired window. An example of this application can be observed in Figure 8.

The mean behaviour of the wavelet transform, considering a 48-frame window of a walking task recorded at 12.5 Hz, gives, as a result, a graph composed of two peaks: one between 0 Hz and 1 Hz, and one between 1 Hz and 4 Hz. The amplitude of these peaks depends on the intensity of the task and is a very strong and consistent discriminant for activity intensity. In particular, the value L_AP is the amplitude of the peak in the range of frequencies 0–1 Hz.

**Figure 9:**
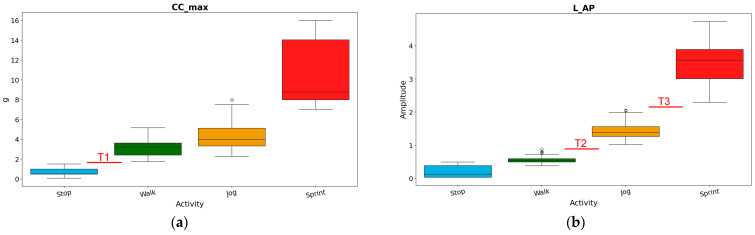
Boxplots of CC maximal amplitude (**a**) and wavelet low-frequency peak amplitude on the AP axis (**b**) for athlete 005 data. T1, T2 and T3 refers to the threshold to differentiate *Stop*, *Walk*, *Jog* and *Sprint*.

After the two features from every 48-frame window were extracted, a statistical analysis of their distributions was considered using boxplots to calculate the three thresholds assigned to the corresponding n-th iteration. In particular, the values of the thresholds were obtained based on the whiskers of the boxplots. The visual representation of the procedure is shown in Figure 9.

With these values, it was possible to determine three subject-specific thresholds (T1, T2, T3) used to discriminate between the four main activities with the following formula:

(4) $$threshold=\frac{upper\;whisker\left(act1\right)-lower\;whisker\left(act2\right)}{2},$$

where *act1* and *act2* represent lower and higher intensity activities, respectively, with *Stop*, *Walk*, *Jog* and *Sprint* being the activities ranked in increasing intensity order. Specifically:

1. T1 (*Stop* vs. *Walk*): determined starting from the upper whisker of *Stop* and the lower whisker of *Walk* of the CC_max;
2. T2 (*Walk* vs. *Jog*): determined starting from the upper whisker of *Walk* and the lower whisker of *Jog* of the L_AP;
3. T3 (*Jog* vs. *Sprint*): determined starting from the upper whisker of *Jog* and the lower whisker of *Sprint* of the L_AP.

**Figure 10:**
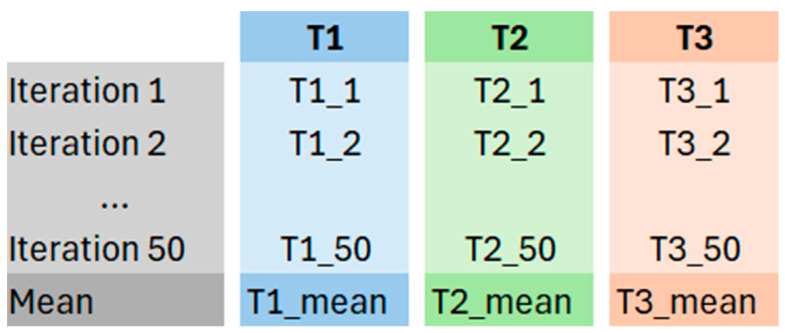
Representation of the threshold storage at the end of the MCCV.

For each n-th iteration of the MCCV, the three thresholds were stored in a specific database, as shown in Figure 10. The final subject-specific thresholds were obtained by calculating the mean value for each parameter across all 50 iterations.

#### 2.8.2. Activity Detection Validation

**Figure 11:**
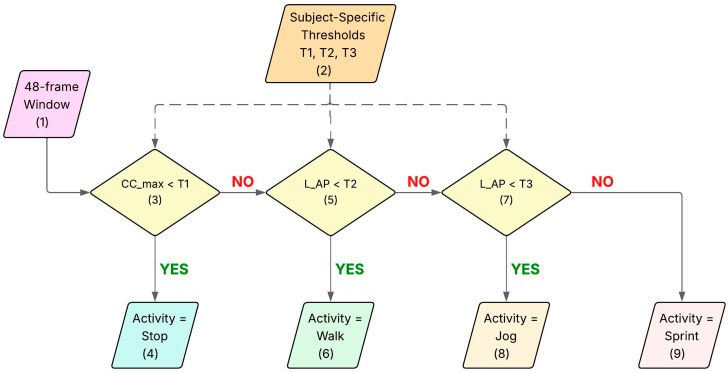
Workflow used to identify the correct activity from the *long-term* acceleration data. The different features used are L_AP, which is the amplitude of the wavelet peak at low frequency in the AP axis, and CC_max, which indicates the maximal acceleration in the CC axis, calculated in the 48-frame windows. T1, T2 and T3 are the thresholds calculated from the whiskers. Each block of the workflow is identified by a number from 1 to 9.

The MCCV was also used to provide a set of outcome measures that describe the performances (error) of the algorithm at a subject-specific level. To do so, during each of the 50 iterations, the sliding window was applied to the corresponding Test Dataset. For every 48-frame window, the two features were extracted. Then, a binary decision tree (Figure 11) was used to assign an activity label to each 24-frame sub window using the thresholds estimated at the n-th iteration of the MCCV on the Training Dataset.

The binary decision tree architecture includes a combination of cascading and parallel decision-making blocks, enabling the flexible and hierarchical processing of sensor data. The detection sequence classified the activities sequentially: *Stop* (Block 11.3), *Walk* (Block 11.5), *Jog* and *Sprint* (Block 11.7) based on the thresholds described in Section 2.8.1.

This gives as a result an *evaluation array*, with the same length as the accelerometer signal, where every frame is assigned a “tag-value” corresponding to an activity: 1 for *Stop*, 2 for *Walk*, 3 for *Jog* and 4 for *Sprint*. Since every MI was previously labelled using the gold standard, by comparing it with the estimated activity class the performances of the algorithm are calculated: a *gold standard array* with the same structure as the *evaluation array* was saved during each iteration, and the two arrays were paired and saved in the database.

The performances of the classifier were tested with a set of well-established statistical metrics (Table 3): Precision, Recall, F1-score, Macro average, Weighted Average and Accuracy) [29]. The following definitions are formulated under the ‘one-vs.-all’ (OvA) evaluation strategy, where each class is treated as the positive class against all others.

In addition, a 4 × 4 confusion matrix was plotted to visualize the True Positives (TPs), True Negatives (TNs), False Negatives (FNs) and False Positives (FPs) for each activity of each athlete. To create this confusion matrix, for each athlete, the arrays containing the labels from the gold standard derived from the 50 iterations of the MCCV were vertically concatenated and the same procedure was executed for the labels predicted by the algorithm. The comparison between these two arrays was used to create the confusion matrix. The best possible outcome was 100% on the diagonal cells and 0% in the other, meaning that the predicted activity always corresponded to the actual ones.

#### 2.8.3. Cadences Estimation

**Figure 12:**
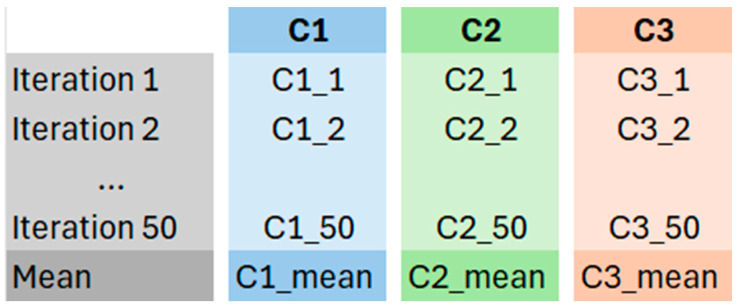
Representation of the cadence storage at the end of the MCCV.

The stride counting algorithm was calibrated for each activity characterized by a cyclic pattern, with the stride considered the primary unit, i.e., *Walk*, *Jog* and *Sprint*. For *Stop* the No. of strides was automatically set to 0. Cadences were calculated using the Train Dataset from each of the 50 iterations of the MCCV and then stored. For each MI, the cadences were estimated using the following formula:

(5) $$Cadence=\frac{No.\;\;of\;strides}{T},$$

where *No*. of strides is the number of strides manually labelled in the MI (gold standard) and *T* is the interval time in minutes, calculated as follows:

(6) $$T=\frac{L}{Fs*60},$$

where *L* is the number of frames of the MIs and Fs is the sampling rate (12.5 Hz). At the end of the 50 iterations, all the cadences were stored in a database (Figure 12), each associated with the corresponding n-th iteration, and the final subject-specific cadences for each activity were calculated as the mean values of the cadences of the 50 iterations.

#### 2.8.4. Stride Counting Validation

The MCCV was also used to validate the stride counting algorithm. During each of the 50 iterations, the duration of each MI in the Test Dataset was extracted, and the number of strides in that MI was estimated by using the cadence estimated during the n-th-iteration of the Train Dataset:

(7) No. strides=cadence∗t,

where t is the time (in minutes) of the current testing MI. Afterwards, the estimated number of strides was stored in the database, alongside the gold standard number of strides manually associated with the testing MI.

At the end of all the iterations, for each athlete and each activity, the total number of strides was obtained by summing the number of strides obtained in each iteration, in short, “detected strides”. The same procedure was repeated for the number of strides extracted from the gold standard, in short, “labelled strides”. The performances of the stride counting algorithm were obtained by calculating the relative error between the total number of labelled strides and the total number of detected strides.

To better categorize each activity, the minimum and maximum speed in km/h for each athlete and activity and mean ± standard deviation for the whole sample were estimated. Speed was assessed from video recordings of the different activities. The tracks contained clearly visible distance markers; by measuring the duration of the video segment in which the athlete traversed a predefined track section, speed was computed as the ratio between the covered distance and the elapsed time.

#### 2.8.5. Overall Workflow Validation

The previous sections describe how the algorithms were evaluated separately, i.e., activity detection and stride counting. However, in the *long-term* application they are expected to be applied subsequently. Therefore, to evaluate the final error of the overall workflow, i.e., the subject-specific performance of the workflow, an extra validation procedure was added to the MCCV analysis.

For each athlete, in each of the 50 MCCV iterations, the number of strides was estimated (Section 2.8.4) within the MIs identified by the activity detection algorithm, using the cadence evaluated during the same iteration (Section 2.8.3). Afterwards, the relative error was estimated considering the number of estimated strides and the number of labelled strides.

### 2.9. Algorithm Application

Once the calibration procedure based on the *short-term* session is completed, the data from the *long-term* session can be analyzed using the two main algorithms, to obtain the training intensities performed by the athletes.

#### 2.9.1. Activity Detection Algorithm

**Figure 13:**
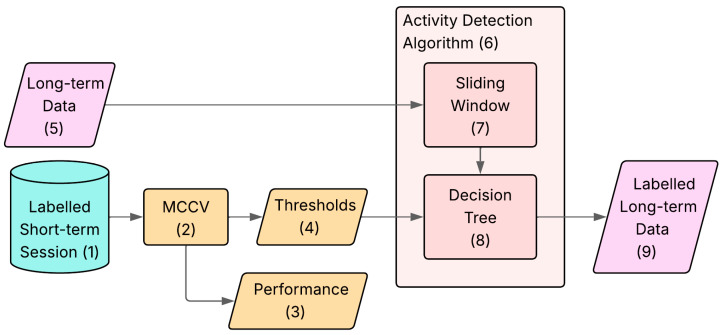
Representation of the workflow of the activity detection algorithm. In light blue (block 1), the data from the *short-term* session. In pink (blocks 5, 9), the data from the *long-term* monitoring. In orange (blocks 2–4), the processes and results from the MCCV. In red (blocks 7–8), the blocks part of the activity detection.

The activity detection algorithm was applied, taking as an input the non-labelled *long-term* session signal and providing the labelled signal as an output. A zoom in of the algorithm is shown in Figure 13.

The sliding window used in Section 2.8.1 was applied to the *long-term* signal (Block 14.7), obtaining the features for every 24-frame window, and using the same binary decision tree from Section 2.8.2 (Block 14.8), a label was assigned to each portion of the signal. As a result, this gave the *long-term* dataset labelled with a resolution of 2 s (Block 14.9), automatically dividing the signal into MIs, which are then saved with a time stamp indicating when they are being performed, giving us a representation of each training session.

#### 2.9.2. Stride Counting Algorithm

**Figure 14:**
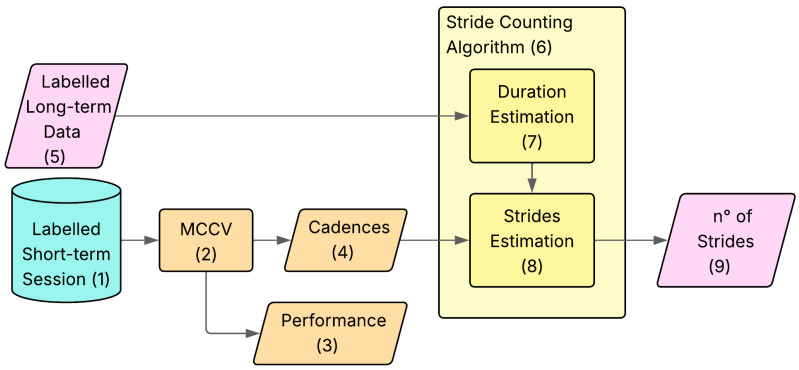
Representation of the workflow of the activity detection algorithm. In light blue (block 1), the data from the *short-term* session. In pink (blocks 5, 9), the data from the *long-term* monitoring. In orange (blocks 2–4), the processes and results from the MCCV. In yellow (blocks 7–8), the blocks part of the stride counting algorithm. Each block of the workflow is identified by a number from 1 to 9.

The stride counting algorithm was applied, taking as an input the labelled *long-term* signal and giving as output the number of strides performed during the whole period of acquisition (Figure 14).

The execution time was derived from the duration of all the MIs associated with each activity (Block 15.7). Subsequently, the number of strides is computed across all the activities (Block 15.9), starting from the Cadence Estimation Block (Block 15.8).

The results were then analyzed to find the daily number of strides performed with the prosthetic device.

### 2.10. Inter-Subject Validation

All the methods described in Section 2.8 and Section 2.9 refer to the calibration, application and validation of the activity detection and stride counting algorithms to a single athlete. However, it is relevant to estimate the overall performance of the algorithm among athletes, i.e., to perform an inter-subject validation.

For the activity detection algorithm, the concatenated arrays created for each athlete with the estimated labels were taken and concatenated in a single array. The same procedure was performed for the corresponding gold standard labels. Using these two arrays, the same metrics described in Section 2.8.2 were calculated.

For the stride counting algorithm, the mean value of strides performed by each athlete was obtained, alongside the mean number of strides counted by the algorithm and the standard deviation (SD). Using these numbers, the same metrics described in Section 2.8.4 were calculated. Finally, for the overall workflow, the same procedure described for the stride counting algorithm was executed using the strides counted with the methods explained in Section 2.8.5.

## 3. Results

### 3.1. Activity Detection Algorithm

**Figure 15:**
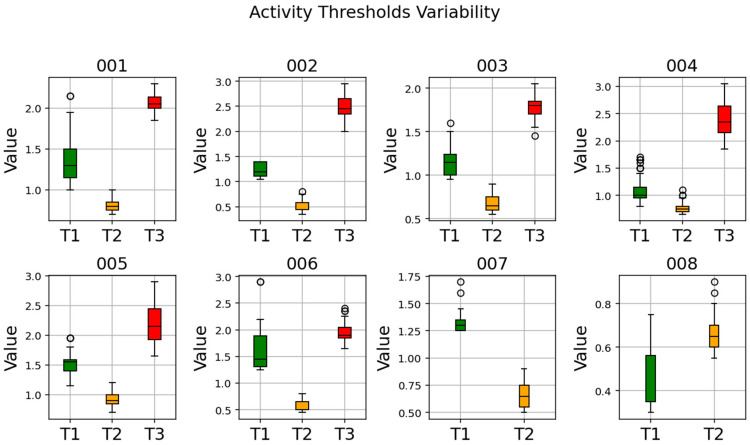
Plots of activity thresholds variability for each athlete in athlete ID ascending order.

**Figure 16:**
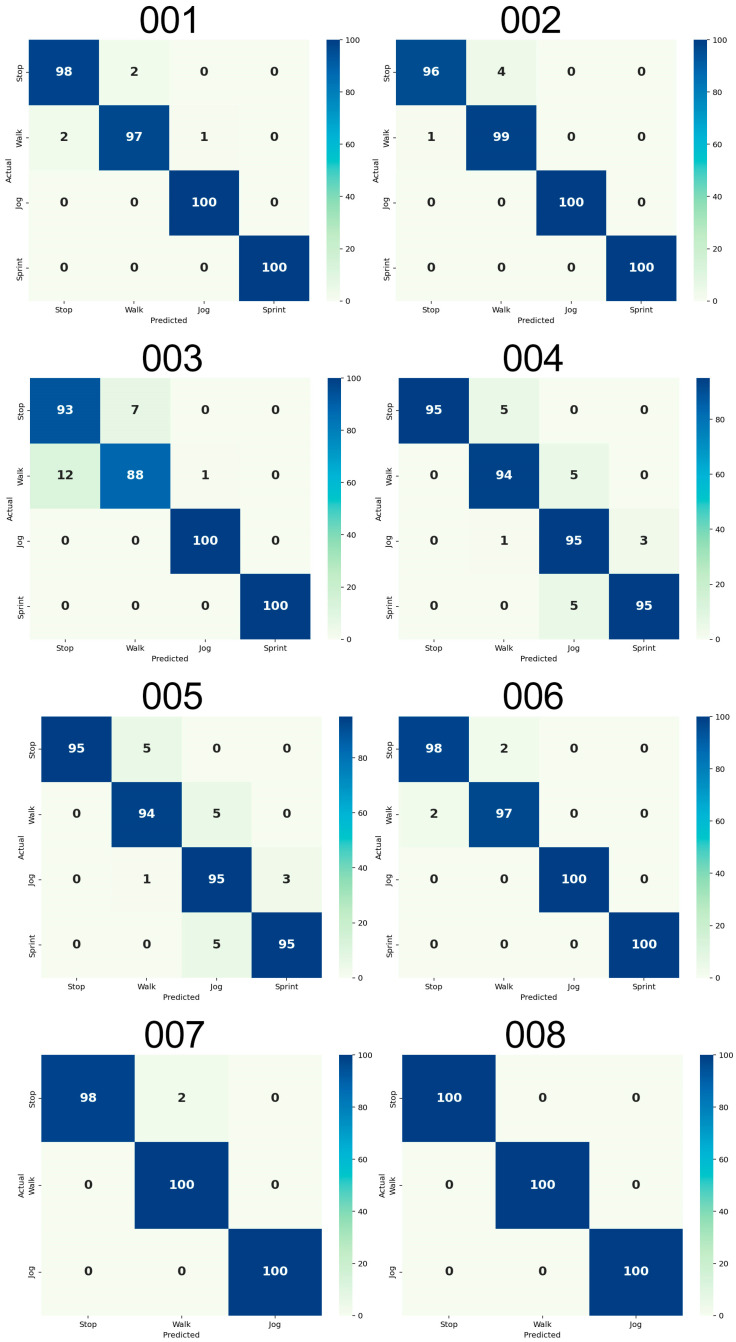
Confusion matrices showing the performances of the algorithm for each athlete.

**Figure 17:**
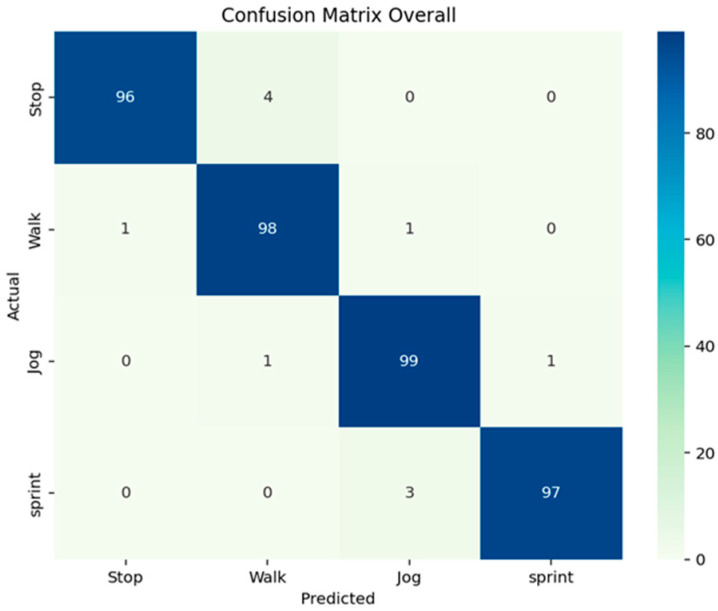
Confusion matrix showing the performances of the overall activity detection algorithm, All the gold standard labels and algorithm-derived labels were concatenated in two arrays and used to create this analysis.

**Figure 18:**
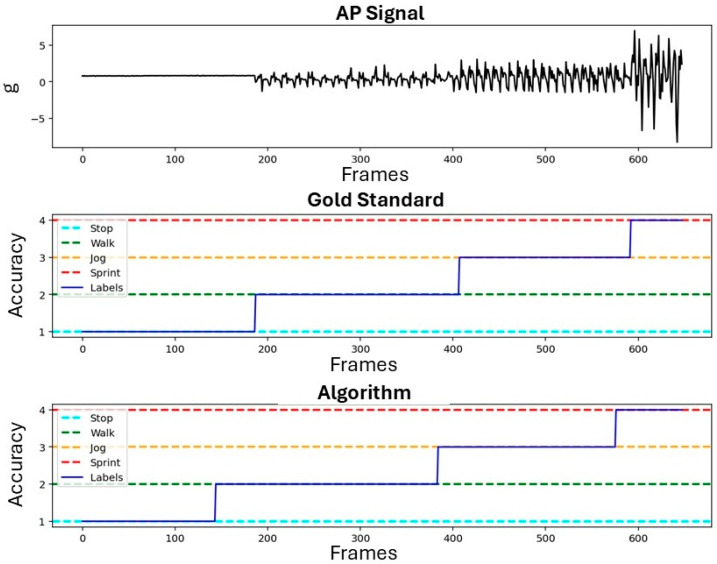
Example of acceleration signal processed by the algorithm. In the first subplot from the top the acceleration signal in the AP axis is shown; the second subplot shows the gold standard activity class; the third subplot shows the activity class detected by the algorithm.

The subject-specific activity thresholds calculated by the MCCV are shown in Table 4. Figure 15 reports the variability of the activity thresholds for each athlete over the 50 iterations of the MCCV. The metrics of the algorithm performance are reported in Table 5. Figure 16 reports the confusion matrices that show the subject-specific performance of the algorithm, Figure 17 reports the confusion matrix regarding the inter-subject performance, and Figure 18 shows an example of the overall classification function of the algorithm: the procedure is applied to a series of four connected MIs, each representing a different activity, following the same intensity order of the MCCV. For athletes performing triathlon (007 and 008), the *Sprint* activity was not recorded, and it will be absent from both the tables and confusion matrices.

### 3.2. Stride Counting Algorithm

**Figure 19:**
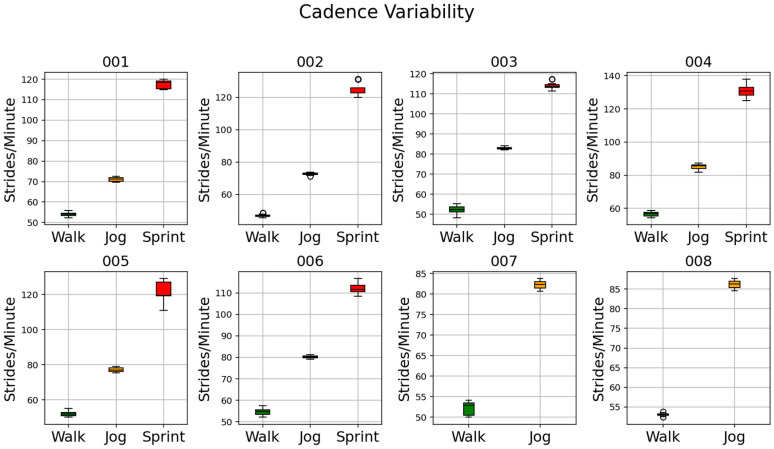
Plots of cadence variability for each athlete in athlete ID ascending order.

The reference cadences, calculated for each athlete as the means of the cadences obtained over 50 iterations of the MCCV, are shown in Table 6. The plots that show the cadence variability over the 50 iterations of the MCCV are reported in Figure 19. Table 7 shows the error percentage of the stride counting algorithm for each athlete, while the bottom row of the table shows the mean values indicating the inter-subject performance. Table 8 reports the speed values obtained for each athlete and activity.

### 3.3. Overall Workflow Performance

The performance of the full workflow is reported in Table 9, indicating the performances of the stride counting algorithm when applied on the activities labelled by the activity detection algorithm, comparing it to the number of strides contained within the gold standard using the MCCV procedure. The table shows the total strides considered to obtain the results and the relative error percentage. The table shows both the subject-specific performance and the inter-subject performance.

### 3.4. Long Term Analysis

**Figure 20:**
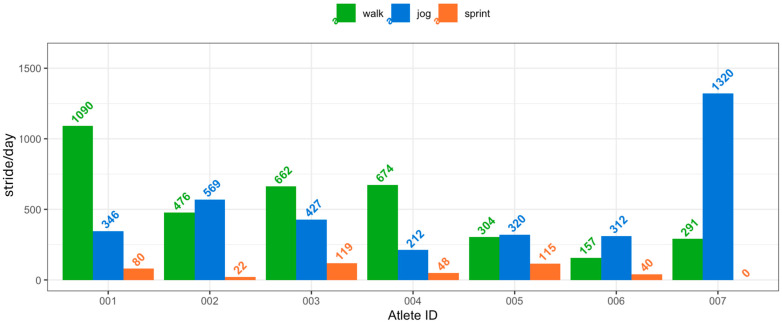
Representation of the daily usage of the prosthetic device by the athletes. In green, the daily walking strides performed. In yellow, the daily jogging strides performed. In red, the daily sprinting strides performed. On the left, the athletes ranging from 001 to 006, who performed all three activities. On the right, athlete 007, as they did not perform sprinting activities. The “a” in the legend is a placeholder for the number of strides reported in the bar plot.

The results of the long-term analysis performed on the signal gathered from around 40 days of data collection before the 2024 Paralympic Games are given in Table 10, and a visual representation of the mean number of daily strides performed by each athlete within each activity is reported in Figure 20. Athlete 007 is a triathlete and does not perform sprinting; therefore, results for this activity are not reported. Athlete 008 is not included in this analysis, as they did not participate in the Paralympic Games and were thus not monitored over the period considered for this study.

For all the athletes, the number of daily strides was calculated based on the days of actual use of the prosthetic device and includes only the activities performed with the prosthesis socket with the AX6 attached, which was also the one used for the competitions.

Table 11 reports the state of the battery after *long-term* monitoring when sensors were returned to the lab.

## 4. Discussion

This study aimed to design and validate an activity detection and a stride counting algorithm, using data from a single, low-sampling frequency triaxial accelerometer, for long-term activity monitoring of track-and-field and triathlon athletes with lower-limb amputations. The instrumentation, performance of each portion of the algorithm and the results of the *long-term* analysis will be commented on separately.

### 4.1. Instrumentation

AX6 managed to record all the athletes across the desired period, and the battery of all the devices was high enough to ensure that a longer monitoring period was available, ranging up to 6 months: on average, after 45 days of recording, the battery life was still in the 80% range. Some intersubject variations were observed in this regard that might be related to different training regimes and starting battery percentage. The routinary application of the sensors for long-term monitoring of Paralympic athletes will provide consolidated insight into the actual battery use.

Despite a simple approach based on the use of adhesive tape, no athlete lost their device during the monitoring period. To better address the variety of the weather conditions and the possibility of triathlon athletes swimming with the device, for future applications we may consider blocking AX6 connections with hydrophobic materials. To ensure better stability of the device, a custom-made case to be embedded on the socket could be developed. Alternatively, the size factor makes AX6 easy to embed inside the prosthetic sockets or in the prosthesis connecting modules (hollow tubes with diameter of 30 mm).

The AX6 sampling frequency was set to 12.5 Hz to accommodate for an extended recording time and considering that the step frequency of elite sprinters was reported to be up to 5.9 Hz [30], which supported our choice from the theoretical perspective for the purpose of activity detection and stride counting. However, other authors also reported low frequency of acceleration signals at the shank to be in the 3 Hz to 8 Hz range and high frequencies reaching 20 Hz even at moderate running speed. [31] Therefore, other analysis outside the specific purpose of activity detection and stride counting might not be possible with a frequency of 12.5 Hz.

### 4.2. Stratified Monte Carlo Cross Validation

The Stratified Monte Carlo Cross Validation (MCCV) [28] was selected to validate the algorithms, as it is a method already present in the literature to assess activity detection algorithms [32]. The decision to consider 50 test–train sets and to then perform a 50% train–test subdivision is a trade-off between accuracy and database size.

The algorithm was designed to classify all long-term data into four activities, namely *Stop*, *Walk*, *Jog*, *Sprint*. This choice was made because athletes use the prostheses on which the AX6 was attached only for competitions and training sessions regarding sprinting or triathlon, and the athletes do not wear the prosthesis during stretching and warm-up exercises performed on the floor. When an unusual activity for physical conditioning is performed with the prosthesis, it is classified by the algorithm as one of the four options. Future works might be intended to expand the range of possible recognized activity, to allow for a greater focus on some part of the physical preparation. However, this was not deemed necessary in this first study, since our focus was mostly on the activities inducing a cyclic loading of the running foot or of the socket.

The set of eight MIs of *Stop*, *Walk* and *Jog* and five for *Sprint* for each athlete was selected for two main reasons. Firstly, to define a viable protocol for the acquisition of *short-term* data that could fit well within a typical athlete training session. Secondly, to collect enough data to complete a subject-specific validation of the thresholds and cadences. It is relevant here to remark that our algorithm does not rely on inter-subject validation only but is capable of providing a subject-specific estimation of the algorithm errors, exploiting the *short-term* session and its division into balanced training and testing datasets. While the results suggest that the number of MIs that we selected for the training and testing datasets is adequate, future studies might concentrate on analyzing the consequences of overall shorter *short-term* sessions. Also, future studies might evaluate the impact on algorithm performances of a “leave-one-out approach” for subject-specific threshold estimation, which has the drawback of unbalancing the testing–training datasets, minimizing the data available for validation, but allows for more variance in the testing database size. Also, future efforts might address estimate inter-subject thresholds, i.e., valid for all athletes, with no need for subject-specific calibrations.

### 4.3. Activity Detection

The performances of this innovative algorithm are much better than the one achieved in the past on lower-limb prostheses users (90%) (LLPUs) [33] and are comparable to performances obtained with complicated and computationally demanding algorithms with gyroscope data on healthy individuals [21].

Specifically, our results show that the method has an overall accuracy of 98%, with the worst performing activity being *Stop* (with an overall accuracy of 96%) and the best-performing activity being *Jog* (with an overall accuracy of 99%). The only case where an athlete showed an activity performing below 90% is *Walk* for athlete 003, reaching an accuracy of 88%, while the best-performing athlete overall was athlete 008, showing an accuracy of 100% for each activity. The different sensor positioning needed for athlete 005 does not seem to influence the performance of the algorithm.

Binary decision tree algorithms based on triaxial accelerometer data for activity recognition have already been tested both on healthy populations [14,15] and on people with transtibial amputations [33,34]. However, the main innovation of this study is the possibility to distinguish activities with a sampling rate of just 12.5 Hz with an extremely high level of accuracy, whereas rates of at least 45 Hz to 100 Hz were previously required. The 12.5 Hz sampling rate leads to huge savings in terms of memory and battery drain, allowing for to data collection up to 6 months, when achieving a month of collection had previously been a challenge.

In particular, the use of the CWT, instead of the 1 Dimension–Continuous Wavelet Transform (1D-DCWT) as used by Sheng et al. [21], made it possible to obtain a classifier based only on accelerometer data (without using a gyroscope that would consume all the battery in a few hours), speeding up the processing time and lowering computational cost. The evaluation of the wavelet mean pattern, rather than the usage of the wavelet transform with other discriminating techniques (e.g., CNN, as used by Nedorubova et al. [35]), is where the innovation lies.

The decision to use the wavelet low peak amplitude in AP and the maximal acceleration amplitude in the CC direction came after a detailed review of many features. Specifically, for each axis we considered the mean of the absolute signal, the mean of the semi-rectified values, the signal energy, the root mean square and the maximal absolute value. For the signal as a whole, we considered its magnitude area and its norm. Our final choice guaranteed that the boxplots’ whiskers of two subsequent activities (in terms of increasing intensity) did not overlap: the upper whisker of the lower-intensity activity was always lower than the lower whisker of the higher-intensity activity. It was verified, on all participants’ dataset, that the wavelet low-frequency peak amplitude boxplots of *Walk* vs. *Jog* and *Jog* vs. *Sprint* did not overlap; the same was found with the maximal amplitude in the CS axis with *Stop* vs. *Walk* boxplots. Non-overlapping boxplots were fundamental to obtaining high level of accuracy in discriminating against different activities. In general, the combination of L_AP and CC_max allowed for the classification of the activities with the lowest amount of features needed, resulting in a simple binary decision tree and a faster signal analysis.

### 4.4. Stride Counting

A stride-counting rather than a more traditional step-counting method was chosen, because as this study was conducted mainly on unilateral prosthesis users, counting each time that the prosthesis touched the floor proved easier.

The errors found for the stride counting algorithm with respect to the gold standard are negligible compared to the more traditional step-counter methods based on higher sampling rates [36,37]. In summary, our study shows that all activities had a mean error inferior to 1% (0.34% for *Walk*, −0.14 for *Jog* and 0.05% for *Sprint*), with *Jog* being the most consistent (variance of 0.41) and *Sprint* being the least inconsistent (variance of 1.28).

The cadence values found for *Walk* are comparable to the values reported in the literature for people with TT and TF amputation (~100 and ~80 steps per minute, respectively, so ~50 and ~40 strides per minute) [38,39,40], even if these studies only considered an everyday prosthesis and not a running-specific prosthesis. Previous studies on running cadence on LLPUs have not been reported. It is known that walking cadence is typical for each healthy individual and maximal variations are lower than 2% [41]. As similar variations were found in this latter study on LLPUs, stride counting from typical cadence was considered a subject-specific reliable stride-counting method.

### 4.5. Overall Workflow Performances

Our study shows that all activities had a mean error comparable to 1% (1.16% for *Walk*, 0.61% for *Jog* and 1.47% for *Sprint*), with *Jog* being the most consistent (variance of 0.91) and *Walk* being the least consistent (variance of 3.04).

The stride-counting errors obtained from the combination of the activity detection and stride counting algorithms are comparable to a traditional step-counter on LLPUs populations.

### 4.6. Long Term Analysis

AX6 sensors were used to collect long-term data from the athletes in the 40 days before the 2024 Paris Paralympic Games. This period is associated with constant training and can possibly provide a worst-case estimation of cyclic loading of running feet and sockets and provide an overall picture of the athlete “habits” under stressful conditions.

As reported in Table 10, every athlete had different training habits. In general, the top-performing athletes (athlete 003 and 005) were also the ones with the most intense training regimes, respectively, with 119 and 115 *Sprint* strides performed daily. While both athletes have a TT amputation, the level of amputation itself did not seem to be a factor in the training regimes, considering that athlete 004 had much less intense training sessions despite having the same level of amputation, performing only 48 strides per day. Athlete 002 received confirmation of participation in the Paralympic Games at a late stage, and because of the uncertainty they were, in general, the one performing the least intense training sessions, with 22 *Sprint* strides daily, being instead the athlete performing the highest number of *Jog* strides per day, with a mean value of 569. Athlete 006 had some personal issues in the period prior to the Paralympic Games, and this influenced their training regimes, showing a small number of strides in each category (40 for *Sprint* and 312 for *Jog*). In general, the discipline seemed to be the biggest factor influencing the training regimes, as the only triathlete (athlete 007) showed a completely different training regime when compared to the ones performed by all other athletes, reaching a mean value of 1320 strides per day.

### 4.7. Limitations

This study presents some limitations that should be acknowledged. First, the sample size was relatively small and restricted to eight elite athletes from the Italian National team, all of whom were running-prosthesis users. The focus on an elite group of Paralympic athletes limits the generalizability of the findings to recreational athletes, users of prosthetic devices in other medical or rehabilitative scenarios or individuals using prosthesis of different models or from different manufacturers. Moreover, the limited availability of athletes for recording sessions resulted in a single acquisition session for most subjects, which may have contributed to a constrained dataset and an increased risk of overfitting.

The algorithms also require a short subject-specific calibration session. Although effective for subject-specific performance, this requirement may hinder scalability in clinical or commercial environments. The absence of a deeper analysis of inter-subject thresholds variation also represents an additional limitation. Furthermore, the low sampling rate, while advantageous for long-term monitoring, restricts the extraction of higher-resolution biomechanical features that could enhance activity characterization. Finally, a direct quantitative comparison with advanced machine learning–based methods (e.g., CNNs, LSTMs) is lacking and should be addressed in future works.

## 5. Conclusions

This study introduced and validated an innovative protocol for sport activity monitoring in Paralympic athletes with TT and TF amputations using running-specific prostheses. The outcome of the study revealed that triaxial accelerometer data with a low sampling rate (12.5 Hz) and CWT are sufficient to accurately classify sport activities (*Stop*, *Walk*, *Jog* and *Sprint*) in LLPUs, with an overall accuracy of 98%. This represents a significant advancement in the field, as the resulting reduction in data size and energy consumption enables *long-term* monitoring in real-world situations for LLPUs in training contexts, enabling a monitoring period that lasts up to 140 days.

In addition, the cadence-based stride-counting approach represents an innovative contribution and could offer valuable insights into the running and walking patterns of both LLPUs and able-bodied individuals, showing an overall stride-counting error inferior to 1% (0.34% for *Walk*, −0.14% for *Jog* and 0.05% for *Sprint*) despite the low sampling rate.

The combination of the activity detection and the stride counting algorithm lays the groundwork for more personalized, efficient, and minimally invasive activity monitoring solutions, showing a stride-counting error comparable to 1% (1.16% for *Walk*, 0.61% for *Jog* and 1.47% for *Sprint*).

Although this study focused on a small group of Paralympic athletes with TT and TF amputations (which is why MCCV and accuracy metrics were preferred to inferential statistics), the methodology could easily be extended to healthy populations, as control, and on a broader range of LLPUs. Accuracy metrics and Monte Carlo Cross Validation were instead used to assess the robustness and generalizability of the algorithm’s performance. With this study, the foundations for more personalized Paralympic training programmes are given, as the high level of accuracy of the algorithms provides useful metrics on the duration of sport activities and on the consequent prosthetic loads. For the same reasons, this approach could be particularly useful for monitoring prosthesis wear over time.

In the near future, we aim to use force platforms to measure the ground reaction forces for every athlete and activity, thus allowing for the definition of accurate load cycles, and apply them to the devices to more accurately evaluate their performances. We also aim at recording more sessions from the athletes in order to have a larger database and more accurately validate the algorithm. We are also planning to apply the procedure in the following years to monitor the performance of the athletes in preparation for the Paralympic Games of Los Angeles in 2028.

## Figures and Tables

**Table 1:** Data for participants’ population: age (years), body mass (kg), level of amputation (TT for transtibial and TF for transfemoral), sex (M for male and F for female), laterality (UNI for unilateral and BI for bilateral), discipline (100 m, 200 m, triathlon), Running foot model (OB: Ottobock, Duderstadt, Germany; OS: Ossur, Reykjavík, Iceland). All athletes with transfemoral amputation used the Ottobock 3S80 prosthetic knee.

Athlete ID	Age (years)	Body Mass (kg)	Level of Amputation	Sex	Laterality	Discipline	Running Foot
001	22	74	TT	M	UNI	100 m, 200 m	OS Xtreme
002	37	78	TF	M	UNI	100 m	OB 1E91
003	19	54	TT	F	UNI	100 m, 200 m	OB 1E90
004	21	62	TT	M	UNI	100 m, 200 m	OS Xtreme
005	33	81	TT	M	UNI	100 m, 200 m	OS Xtreme
006	23	54	TF	F	UNI	100 m	OB 1E91
007	27	57	TT	F	BI	Triathlon	OB 1E90
008	53	68	TF	M	UNI	Triathlon	OB 1E91
Mean ± std	29 ± 11	66 ± 11	5 TT, 3 TF	3F, 5M	1 BI, 7 UNI	6 track, 2 Triathlon	

**Table 2:** Summary of the selected features to discriminate between different activities.

Activities to Discriminate	Discriminating Features	Feature Acronym
*Stop* vs. remaining activities	Maximal acceleration amplitude in the CC axis	CC_max
*Walk* vs. remaining activities	Amplitude of the wavelet peak at Low frequency (0–1 Hz) for the AP axis	L_AP
*Jog* vs. *Sprint*		

**Table 3:** Formulas used to calculate precision, recall and F1-score, macro and weighted average and accuracy of the activity detection algorithm. TP = true positive, FP = false positive, FN = false negative and TN = true negative. Metric refers to precision, recall and F1 calculated for each activity class. Support indicates the number of samples for each class.

Outcome Parameter	Formula
Precision (for each activity class)	${Precision}_{i}=\frac{{TP}_{i}}{{TP}_{i}+{FP}_{i}}$
Recall (for each activity class)	${Recall}_{i}=\frac{{TP}_{i}}{{TP}_{i}+{FN}_{i}}$
F1-score (for each activity class)	${F1}_{i}=2*\frac{{Precision}_{i}*{Recall}_{i}}{{Precision}_{i}+{Recall}_{i}}$
Macro average (for each metric)	$Macro\;avg=\frac{1}{N}\sum_{i=1}^{N}{Metric}_{i}$
Weighted average (for each metric)	$Weighted\;avg=\frac{\sum_{i=1}^{N}{(Metric}_{i}*{Support}_{i})}{\sum_{i=1}^{N}{Support}_{i}}$
Accuracy (overall)	$Accuracy=\frac{\sum_{i=1}^{N}{TP}_{i}}{\sum_{i=1}^{N}\left({TP}_{i}+{FP}_{i}+{FN}_{i}+{TN}_{i}\right)}$

**Table 4:** Mean thresholds for each athlete and activity, calculated from the Train Database. Thresholds 1, 2 and 3 refer, respectively, to the threshold of CC maximal acceleration amplitude used to differentiate between *Stop* and *Walk*, to the threshold amplitude of the wavelet transform in the AP axis used to differentiate between *Walk* and *Jog* and to the threshold amplitude of the wavelet transform in the AP axis to differentiate between *Jog* and *Sprint*.

Athlete ID	Threshold 1 (g)	Threshold 2 (g)	Threshold 3 (g)
001	1.39	0.8	2.07
002	1.24	0.51	2.49
003	1.16	0.69	1.79
004	1.09	0.78	2.40
005	1.51	0.91	2.20
006	1.62	0.57	1.95
007	1.32	0.67	-
008	0.44	0.66	-
mean ± std	1.22 ± 0.36	0.70 ± 0.13	2.15 ± 0.27

**Table 5:** Values of precision (%), recall (%) and F1-score (%) calculated on the total number of labelled MIs (support) for the all-athletes database for each activity (*Stop*, *Walk*, *Jog* and *Sprint*).

	Precision %	Recall %	f1-Score %	Support	Accuracy %
*Stop*	98	96	97	296,473	-
*Walk*	97	98	97	406,888	-
*Jog*	98	99	98	266,548	-
*Sprint*	97	97	97	73,911	-
Overall	-	-	-	1,047,020	98
macro avg	98	97	98	1,047,020	-
weight avg	97	97	97	1,047,020	-

**Table 6:** Cadence (strides per minute) for each athlete and activity calculated from the manually labelled Train Database.

Athlete ID	Walk Cadence (Strides Per Minute)	Jog Cadence (Strides Per Minute)	Sprint Cadence (Strides Per Minute)
001	54	71	117
002	47	73	125
003	52	83	114
004	57	85	131
005	52	77	121
006	55	80	112
007	52	82	/
008	53	86	/
mean ± std	53 ± 3	80 ± 6	120 ± 7

**Table 7:** Total of strides and relative error percentage for each athlete and for the full participant database for the stride counting algorithm in the three activities (*Walk*, *Jog*, *Sprint*) on the gold standard (manually labelled data).

Athlete ID	Total Strides Walk	Error Gold Standard Walk (%)	Total Strides Jog	Error Gold Standard Jog (%)	Total Strides Sprint	Error Gold Standard Sprint (%)
001	3414	0.43	2895	0.01	1257	0.42
002	2801	−0.59	4297	−0.33	2135	−2.57
003	2433	1.51	3875	−0.07	1929	0.22
004	6811	0.04	5379	−1	1668	0.32
005	2879	−0.47	2750	−0.08	2529	1.13
006	4599	0.38	3199	−0.03	2674	0.16
007	2876	1.57	3088	0.45	-	-
008	3007	−0.13	2868	−0.03	-	-
mean ± std	3603 ± 1451	0.34 ± 0.82	3544 ± 916	−0.14 ± 0.41	2032 ± 532	0.05 ± 1.28

**Table 8:** Min and max speed of *Walk*, *Jog* and *Sprint* in km/h for each athlete and mean and standard deviation (std) for the whole database.

Athlete ID	Min Walk (km/h)	Max Walk (km/h)	Min Jog (km/h)	Max Jog (km/h)	Min Sprint (km/h)	Max Sprint (km/h)
001	3.6	4.5	8	10.3	28	36
002	4	5.1	7.2	15	18	24
003	3.2	4.8	6.3	7.6	19.2	26
004	3.4	5.2	7.2	12	18	24
005	3.6	5.3	8	14	30	38
006	3	4.5	8	10	19.2	24
007	4	5.1	9	12	-	-
008	4.32	5.1	9.5	12	-	-
mean ± std	3.64 ± 0.44	4.95 ± 0.31	7.90 ± 1.02	11.61 ± 2.33	22.07 ± 5.43	28.67 ± 6.53

**Table 9:** Total of strides and relative error percentage for each athlete and for the full participant database for the stride counting algorithm in the three activities (*Walk*, *Jog*, *Spring*) on the gold standard (manually labelled data).

Athlete ID	Total Strides Walk	Error Gold Standard Walk (%)	Total Strides Jog	Error Gold Standard Jog (%)	Total Strides Sprint	Error Gold Standard Sprint (%)
001	3414	0.82	2895	0.93	1257	0.42
002	2801	2.25	4297	1.41	2135	2.57
003	2433	−4.58	3875	0.65	1929	0.03
004	6811	1.81	5379	0.81	1668	5.91
005	2879	2.75	2750	1.87	2529	3.33
006	4599	−2.13	3199	0.13	2674	0.16
007	2876	4.02	3088	0.45	-	-
008	3007	2.7	2868	−1.24	-	-
mean ± std	3603 ± 1451	1.16 ± 3.04	3544 ± 916	0.61 ± 0.91	2032 ± 532	1.47 ± 2.62

**Table 10:** Results of the *long-term* analysis. ‘Total Days’ indicates the number of days the sensor was kept on the prosthesis of the athlete and recording. ‘Days of Use’ indicates the number of days where a meaningful signal was found. The time for the three activities indicates the full amount of time a certain activity has been performed, as identified by the algorithm. The number of strides indicates how many strides were performed for each activity by multiplying the time for the cadence, and in brackets, the daily strides are indicated, which represent the mean number of strides performed in each day of actual use.

Athlete ID	Total Days	Days of Use	Walk Time	Jog Time	Sprint Time	Walk Strides	Jog Strides	Sprint Strides
001	72	42	14 h 8 m 1 s	3 h 24 m 57 s	0 h 28 m 57 s	45,794 (1090/d)	14,552 (346/d)	3388 (80/d)
002	51	30	5 h 3 m 54 s	3 h 51 m 55 s	0 h 5 m 26 s	14,283 (476/d)	17,076 (569/d)	680 (22/d)
003	41	26	5 h 31 m 27 s	2 h 14 m 0 s	0 h 27 m 19 s	17,236 (662/d)	11,123 (427/d)	3115 (119/d)
004	40	35	6 h 54 m 0 s	1 h 27 m 33 s	0 h 13 m 3 s	23,599 (674/d)	7442 (212/d)	1710 (48/d)
005	40	30	2 h 55 m 52 s	2 h 5 m 3 s	0 h 28 m 30 s	9145 (304/d)	9629 (320/d)	3450 (115/d)
006	38	16	0 h 45 m 45 s	1 h 2 m 33 s	0 h 05 m 43 s	2517 (157/d)	5005 (312/d)	642 (40/d)
007	34	21	1 h 57 m 43 s	5 h 38 m 10 s	-	6122 (291/d)	27,730 (1320/d)	\

**Table 11:** Battery life of Ax6, including the days of activity, the sensor battery at the start of recording, the sensor battery as it reached the lab after the *long-term* analysis and the battery variation.

Athlete	Days of Recording	Starting Battery (%)	Final Battery (%)	Battery Variation (%)
001	72	92	70	22
002	51	89	75	14
003	41	100	88	12
004	40	90	85	5
005	40	89	81	8
006	38	90	82	8
007	34	89	84	5
mean± std	45 ± 13	91 ± 4	81 ± 6	11 ± 6

## Data Availability

Research data are not shared due to participant privacy. Data are available from the corresponding author upon reasonable request.

## Abbreviations

The following abbreviations are used in this manuscript:

IMU	Inertial Measurement Unit
TT	Transtibial
TF	Transfemoral
GUI	Graphical User Interface
AP	Antero-Posterior
CC	Cranio-Caudal
ML	Medio-Lateral
MEMs	Micro-Electro-Mechanical Systems
MI	Motion Interval
MCCV	Monte Carlo Cross Validation
CC_max	Cranio-Caudal Maximal Value
L_AP	Low frequency wavelet peak in the AP axis
CWT	Continuous Wavelet Transform
Q1	First Quartile
Q3	Third Quartile
IQR	Inter Quartile Range
OvA	One vs. All
TP	True Positive
TN	True Negative
FN	False Negative
FP	False Positive
SD	Standard Deviation
LLPU	Lower-Limb Prosthesis User

## References

[1] Bragaru M., Dekker R., Geertzen J.H.B., Dijkstra P.U. (2011). Amputees and Sports: A Systematic Review. Sports Med..

[2] Matthews D., Sukeik M., Haddad F. (2014). Return to sport following amputation. J. Sports Med. Phys. Fit..

[3] Laferrier J.Z., Parente M., Felmlee D. (2024). Return to Sport, Exercise, and Recreation (SER) Following Amputation. Curr. Phys. Med. Rehabil. Rep..

[4] Legg D. (2018). Paralympic Games: History and Legacy of a Global Movement. Phys. Med. Rehabil. Clin. N. Am..

[5] Gold J.R., Gold M.M. (2007). Access for all: The rise of the Paralympic Games. J. R. Soc. Promot. Health.

[6] Herzog W. (2024). The Paris 2024 Olympic and Paralympic Games. J. Sport Health Sci..

[7] Migliore G.L., Petrone N., Hobara H., Nagahara R., Miyashiro K., Costa G.F., Gri A., Cutti A.G. (2021). Innovative alignment of sprinting prostheses for persons with transfemoral amputation: Exploratory study on a gold medal Paralympic athlete. Prosthet. Orthot. Int..

[8] Petrone N., Costa G., Foscan G., Gri A., Mazzanti L., Migliore G., Cutti A.G. (2020). Development of Instrumented Running Prosthetic Feet for the Collection of Track Loads on Elite Athletes. Sensors.

[9] Gariboldi F., Scapinello M., Migliore G.L., Cutti A.G., Petrone N. (2025). Structural evaluation of lower-limb prosthetic sockets for running—Part 1: Design, implementation and first assessment of an innovative test bench. Results Eng..

[10] Gariboldi F., Scapinello M., Migliore G.L., Petrone N., Teti G., Cutti A.G. (2025). Structural evaluation of lower-limb prosthetic sockets for running—Part 2: Exploratory application of an innovative test bench to various socket designs. Results Eng..

[11] Hutabarat Y., Owaki D., Hayashibe M. Seamless Temporal Gait Evaluation during Walking and Running Using Two IMU Sensors. Proceedings of the 2021 43rd Annual International Conference of the IEEE Engineering in Medicine & Biology Society (EMBC).

[12] Little C., Lee J.B., James D.A., Davison K. (2013). An evaluation of inertial sensor technology in the discrimination of human gait. J. Sports Sci..

[13] Yu S., Yang J., Huang T.-H., Zhu J., Visco C.J., Hameed F., Stein J., Zhou X., Su H. (2023). Artificial Neural Network-Based Activities Classification, Gait Phase Estimation, and Prediction. Ann. Biomed. Eng..

[14] Mathie M.J., Coster A.C.F., Lovell N.H., Celler B.G. (2003). Detection of daily physical activities using a triaxial accelerometer. Med. Biol. Eng. Comput..

[15] Mathie M.J., Celler B.G., Lovell N.H., Coster A.C.F. (2004). Classification of basic daily movements using a triaxial accelerometer. Med. Biol. Eng. Comput..

[16] Arch E.S., Sions J.M., Horne J., Bodt B.A. (2018). Step count accuracy of StepWatch and FitBit One^TM^ among individuals with a unilateral transtibial amputation. Prosthet. Orthot. Int..

[17] Stevens W.R., Barrett C., Jeans K.A. (2024). Comparison of three device generations of the StepWatch Activity Monitor: Analysis of model version agreement in pediatric and adult independent ambulators. Front. Sports Act. Living.

[18] Orendurff M.S., Kobayashi T., Villarosa C.Q., Coleman K.L., Boone D.A. (2016). Comparison of a computerized algorithm and prosthetists’ judgment in rating functional levels based on daily step activity in transtibial amputees. J. Rehabil. Assist. Technol. Eng..

[19] Mellema M., Gjøvaag T. (2022). Reported Outcome Measures in Studies of Real-World Ambulation in People with a Lower Limb Amputation: A Scoping Review. Sensors.

[20] Smith J.D., Guerra G. (2021). Quantifying step count and oxygen consumption with portable technology during the 2-min walk test in people with lower limb amputation. Sensors.

[21] Sheng M., Wang W.-J., Tong T.-T., Yang Y.-Y., Chen H.-L., Su B.-Y. (2021). Motion Intent Recognition in Intelligent Lower Limb Prosthesis Using One-Dimensional Dual-Tree Complex Wavelet Transforms. Comput. Intell. Neurosci..

[22] Young A.J., Hargrove L.J. (2016). A Classification Method for User-Independent Intent Recognition for Transfemoral Amputees Using Powered Lower Limb Prostheses. IEEE Trans. Neural Syst. Rehabil. Eng..

[23] Zheng E., Wang Q. (2017). Noncontact Capacitive Sensing-Based Locomotion Transition Recognition for Amputees With Robotic Transtibial Prostheses. IEEE Trans. Neural Syst. Rehabil. Eng..

[24] Gonzales-Huisa O.A., Oshiro G., Abarca V.E., Chavez-Echajaya J.G., Elias D.A. (2023). EMG and IMU Data Fusion for Locomotion Mode Classification in Transtibial Amputees. Prosthesis.

[25] Marcos Mazon D., Groefsema M., Schomaker L.R.B., Carloni R. (2022). IMU-Based Classification of Locomotion Modes, Transitions, and Gait Phases with Convolutional Recurrent Neural Networks. Sensors.

[26] Demeco A., Frizziero A., Nuresi C., Buccino G., Pisani F., Martini C., Foresti R., Costantino C. (2023). Gait Alteration in Individual with Limb Loss: The Role of Inertial Sensors. Sensors.

[27] Axivity Ltd. *AX6 Data Sheet—Update 20230418*; Axivity Ltd.: Newcastle upon Tyne, UK, 2023. https://axivity.com/downloads/ax6.

[28] Xu Q.-S., Liang Y.-Z. (2001). Monte Carlo cross validation. Chemom. Intell. Lab. Syst..

[29] Sokolova M., Lapalme G. (2009). A systematic analysis of performance measures for classification tasks. Inf. Process. Manag..

[30] Ae M. (2017). Sprint Running: Running at Maximum Speed. Handbook of Human Motion.

[31] Xiang L., Gu Y., Rong M., Gao Z., Yang T., Wang A., Shim V., Fernandez J. (2022). Shock Acceleration and Attenuation during Running with Minimalist and Maximalist Shoes: A Time- and Frequency-Domain Analysis of Tibial Acceleration. Bioengineering.

[32] Alagoz C. (2024). Comparative Analysis of XGBoost and Minirocket Algortihms for Human Activity Recognition. arXiv.

[33] Bussmann H.B., Reuvekamp P.J., Veltink P.H., Martens W.L., Stam H.J. (1998). Validity and Reliability of Measurements Obtained With an “Activity Monitor” in People with and Without a Transtibial Amputation. Phys. Ther..

[34] Redfield M.T., Cagle J.C., Hafner B.J., Sanders J.E. (2013). Classifying prosthetic use via accelerometry in persons with transtibial amputations. J. Rehabil. Res. Dev..

[35] Nedorubova A., Kadyrova A., Khlyupin A. (2021). Human Activity Recognition using Continuous Wavelet Transform and Convolutional Neural Networks. arXiv.

[36] Bastas G., Fleck J.J., Peters R.A., Zelik K.E. (2018). IMU-based gait analysis in lower limb prosthesis users: Comparison of step demarcation algorithms. Gait Posture.

[37] Rigot S.K., Maronati R., Lettenberger A., O’Brien M.K., Alamdari K., Hoppe-Ludwig S., McGuire M., Looft J.M., Wacek A., Cave J. (2024). Validation of Proprietary and Novel Step-counting Algorithms for Individuals Ambulating With a Lower Limb Prosthesis. Arch. Phys. Med. Rehabil..

[38] Schuett D.J., Wyatt M.P., Kingsbury T., Thesing N., Dromsky D.M., Kuhn K.M. (2019). Are Gait Parameters for Through-knee Amputees Different from Matched Transfemoral Amputees?. Clin. Orthop..

[39] Highsmith M.J., Schulz B.W., Hart-Hughes S., Latlief G.A., Phillips S.L. (2010). Differences in the Spatiotemporal Parameters of Transtibial and Transfemoral Amputee Gait. J. Prosthet. Orthot..

[40] Yang J.R., Yang H.S., Ahn D.H., Ahn D.Y., Sim W.S., Yang H.-E. (2018). Differences in Gait Patterns of Unilateral Transtibial Amputees with Two Types of Energy Storing Prosthetic Feet. Ann. Rehabil. Med..

[41] Terrier P., Schutz Y. (2003). Variability of gait patterns during unconstrained walking assessed by satellite positioning (GPS). Eur. J. Appl. Physiol..

